# Histone modifications and their roles in macrophage-mediated inflammation: a new target for diabetic wound healing

**DOI:** 10.3389/fimmu.2024.1450440

**Published:** 2024-08-20

**Authors:** Jing Wang, Jiawei Feng, Yiming Ni, Yuqing Wang, Ting Zhang, Yemin Cao, Mingmei Zhou, Cheng Zhao

**Affiliations:** ^1^ Shanghai Traditional Chinese Medicine Integrated Hospital, Shanghai University of Traditional Chinese Medicine, Shanghai, China; ^2^ Institute of Interdisciplinary Integrative Medicine Research, Shanghai University of Traditional Chinese Medicine, Shanghai, China

**Keywords:** diabetic wounds, histone modifications, histone methylation, histone acetylation, histone-modifying enzymes, macrophage

## Abstract

Impaired wound healing is one of the main clinical complications of type 2 diabetes (T2D) and a major cause of lower limb amputation. Diabetic wounds exhibit a sustained inflammatory state, and reducing inflammation is crucial to diabetic wounds management. Macrophages are key regulators in wound healing, and their dysfunction would cause exacerbated inflammation and poor healing in diabetic wounds. Gene regulation caused by histone modifications can affect macrophage phenotype and function during diabetic wound healing. Recent studies have revealed that targeting histone-modifying enzymes in a local, macrophage-specific manner can reduce inflammatory responses and improve diabetic wound healing. This article will review the significance of macrophage phenotype and function in wound healing, as well as illustrate how histone modifications affect macrophage polarization in diabetic wounds. Targeting macrophage phenotype with histone-modifying enzymes may provide novel therapeutic strategies for the treatment of diabetic wound healing.

## Highlights

- Macrophages have different phenotypes during the formation and development of diabetic wounds, and these phenotypes can be modulated by histone modifications.
- Histone modification patterns are altered in diabetic macrophages, driving the generation of pro-inflammatory macrophages and creating a pro-inflammatory feedback loop that leads to uncontrolled inflammation.
- The regulatory enzymes involved in histone modifications affecting macrophage phenotype may provide a new therapeutic strategy for wound healing.

## 1 Introduction

It has been reported that diabetic wounds affect roughly 18.6 million individuals globally each year, with severe instances leading to lower limb amputation and a significant socioeconomic burden (1). Diabetic wounds are hard to heal for a wide variety of causes, and one of the primary causes is chronic inflammation. Macrophage phenotypic imbalance can cause chronic inflammation in diabetic wounds (2). Uncontrolled inflammation and abnormal macrophage phenotypic alterations are major impediments to diabetic wound healing.

**Figure 1:**
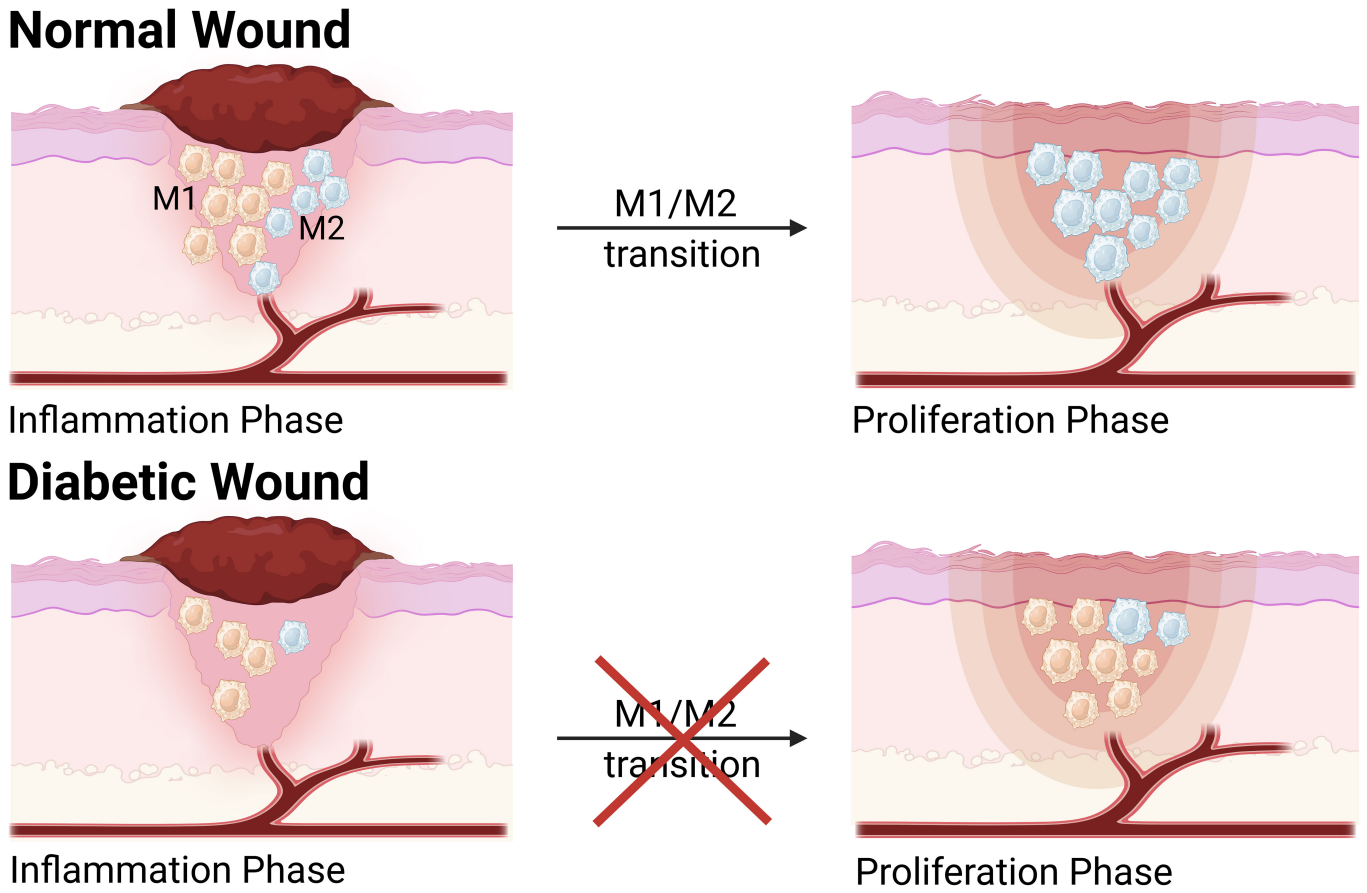
The transition of M1 to M2 phenotype in normal wound and diabetic wound. Compared to normal wound, macrophage polarization is dysregulated in the diabetic wound, which hinders the transition of the diabetic wound healing process from the inflammatory phase to the proliferative phase. During the inflammatory phase, the levels of M1 and M2 macrophages are significantly reduced in diabetic wound. During the proliferative phase, the population of M1 macrophages in diabetic wounds is considerably higher than that in normal wounds, while M2 macrophages remain at a relatively low level. This figure was created with the aid of Biorender (https://biorender.com/).

Macrophages are crucial immune cells actively involved in wound regeneration and serve as critical regulators of wound healing and tissue regeneration, with a high plasticity and continually changing phenotype (3). As wound healing develops following tissue damage, macrophages exhibit considerable phenotypic changes in response to external stimuli. During the typical wound healing process, macrophages display a pro-inflammatory phenotype (M1) in the early stages of tissue repair before transitioning to a pro-healing, anti-inflammatory phenotype (M2) in the later stages, which is referred to as macrophage polarization (4). However, in diabetic wounds, macrophage transition from a pro-inflammatory to an anti-inflammatory state does not occur, leading to impaired wound healing and prolonged inflammatory states (5–7) (**Figure 1**).

Genetic and environmental factors influence diabetic wounds, and their pathogenesis may involve epigenetic alterations, particularly histone modifications. Chromatin structure remodeling induced by histone modifications is crucial for regulating eukaryote gene expression. Previous research has shown that the macrophage phenotype shift during diabetic wound healing can be influenced by gene regulation by histone modifications (8–10). The histone methyltransferase Setdb2 suppresses gene expression by trimethylation of H3K9. Kimball et al. discovered that Setdb2 enhances the repressive H3K9me3 in normal wound macrophages, which leads to decreased interleukins-1β (IL-1β) and tumor necrosis factor-α (TNF-α) transcription on day 5, a critical period during wound healing when macrophages change from an inflammatory to a reparative state (10). The pro-inflammatory macrophage phenotype is promoted by altered histone methylation and histone acetylation patterns in diabetic wound macrophages compared to normal wounds. Recent research has shown that targeting the histone demethylase jumonji domain containing-3 (Jmjd3) in a local, macrophage-specific way can reduce inflammation and improve diabetic wound healing (11). This article reviews macrophages’ role in wound healing and how their phenotype and function are disrupted in diabetic wounds. We will also present new discoveries that shed light on the mechanisms underlying how histone modifications control macrophage phenotype and function in an inflammatory environment. Targeting histone-modifying enzymes to alter macrophage phenotype may offer a novel therapeutic approach for the treatment of diabetic wounds.

## 2 Macrophage phenotype and function in wound healing

**Figure 2:**
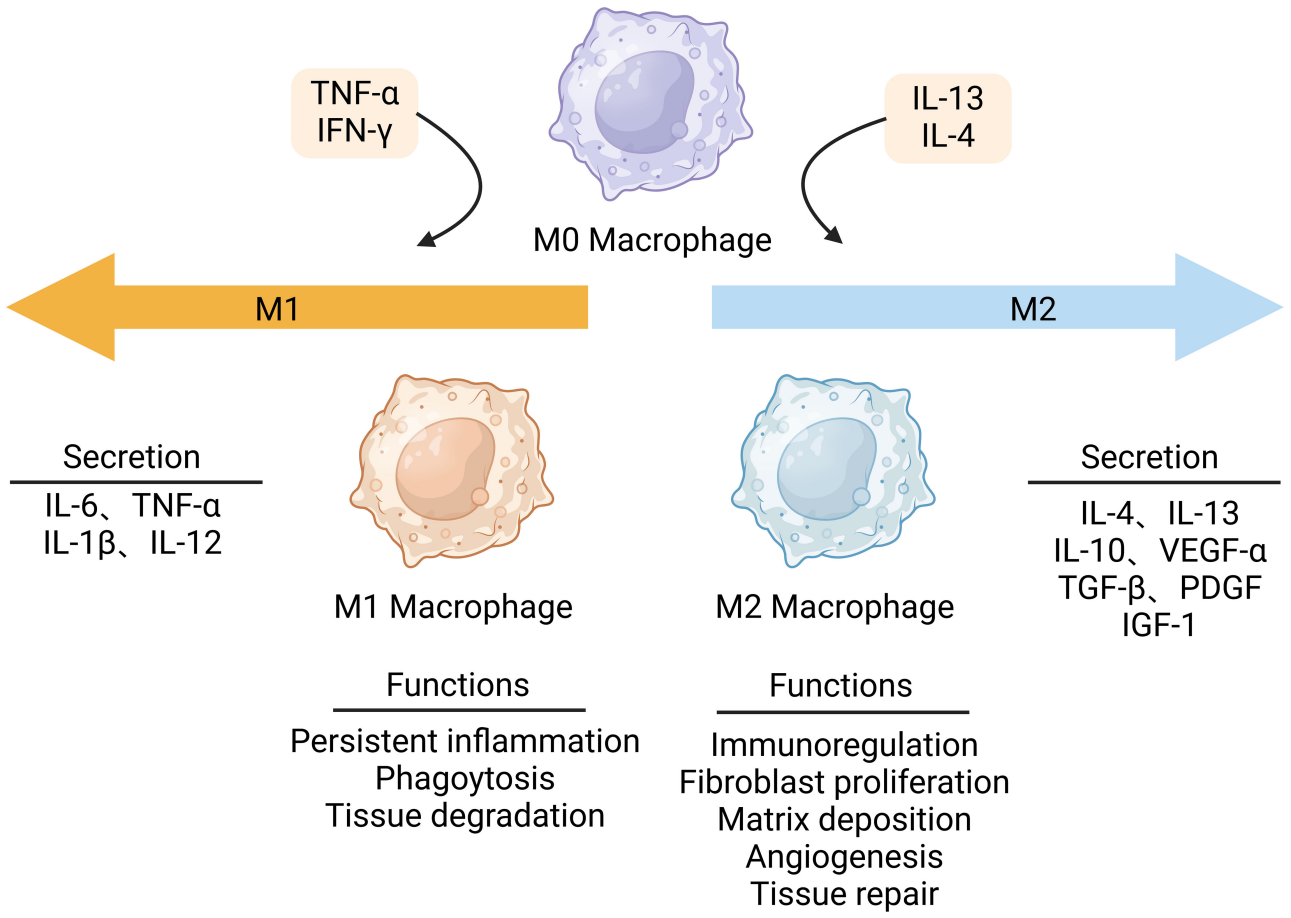
Macrophage polarization in wound healing. Macrophages can polarize to M1 pro-inflammatory phenotype or M2 anti-inflammatory phenotype. Stimulated by interferon γ (IFN-γ), lipopolysaccharide (LPS), and tumor necrosis factor-α (TNF-α), M1 macrophages dominate in the early stages of wound healing. They produce pro-inflammatory mediators that promote the initial inflammation response. When the wound begins to repair, M2 macrophages are activated by IL-4 or IL-13 to produce anti-inflammatory factors. This figure was created with the aid of Biorender (https://biorender.com/).

Wound healing is an intricate physiological process that involves various cellular and molecular interactions. Plenty of evidence indicates that macrophages are key regulators in wound healing, with their phenotype and function dynamically transforming as a reply to external stimuli. In various wound environments, macrophages undergo dynamic changes in phenotype and function under the stimulation of different cytokines, which is referred to as macrophage polarization (12). After skin injury, monocytes gather at the wound site and differentiate into macrophages (13). Macrophages at the site of infiltrating wounds are activated in the presence of stimulants, including interferon-γ (IFN-γ), TNF-α, and lipopolysaccharide (LPS) (14), resulting in M1 phenotype. M1 macrophages predominate during the initial phases of wound healing, expressing pro-inflammatory cytokines, such as interleukins-6 (IL-6), TNF-α, and IL-1β, and other pro-inflammatory cytokines, exhibiting pro-inflammatory properties (15). Pro-inflammatory macrophages (M1) have strong microbicidal properties and are able to identify and phagocytose pathogens. Apart from bactericidal effects, the interleukins-12 (IL-12) secreted by the M1 macrophages can activate the T helper 1 (Th1) cells to trigger an adaptive immune response (16). During normal wound healing, in order to minimize inflammatory-induced tissue damage, the pro-inflammatory macrophage phenotype (M1) is converted to the anti-inflammatory macrophage phenotype, which is also called alternatively activated M2 macrophage phenotype. Over time, M2 macrophages are stimulated by interleukins-4 (IL-4) and interleukins-13 (IL-13) and gradually dominate, secreting anti-inflammatory cytokines such as interleukins-10 (IL-10) to decrease the inflammation response, as well as growth factors like vascular endothelial growth factors-α (VEGF-α), transforming growth factor-β (TGF-β), platelet-derived growth factors (PDGF) and insulin-like growth factor-1 (IGF-1) to promote angiogenesis and cell proliferation (**Figure 2**) (17). During the initial stage of wound repair, neutrophils kill invading pathogens by releasing toxic particles, generating oxidative bursts, initiating phagocytosis, and producing neutrophil extracellular traps (NETs) (18). Elevated levels of proteases generated from neutrophils can lead to chronic inflammation (19). Free oxygen radicals released by neutrophils damage normal tissues (20). Once neutrophils have completed phagocytosis, they need to be removed from the wound site to prevent chronic inflammation (21). In addition, macrophages perform efferocytosis, which is essential for the removal of depleted neutrophils during wound healing (22). The production of IL-10 by M2 macrophages has a role in inducing neutrophil death and promoting collagen deposition. This procedure facilitates the elimination of neutrophils, inhibits inflammation, and promotes tissue regeneration (23). Afterward, macrophages eliminate the apoptotic neutrophils through phagocytosis, preventing further tissue injury and the accumulation of collagen in scar tissue (24).

## 3 Macrophage polarization dysregulation in diabetic wounds

Macrophage polarization is vital for the progression and inflammation resolution during the process of wound healing. In diabetic wounds, however, inflammation resolution failed to be achieved, and research found that this was associated with dysregulated macrophage polarization (6, 25). Macrophage polarization is essential for commencing the initial inflammatory phase and facilitating the conversion from a pro-inflammatory environment to an anti-inflammatory environment. During the normal wound healing process, macrophages initially display a pro-inflammatory phenotype (M1) in the early stages of tissue repair and then shift to an anti-inflammatory phenotype (M2) in the later stages (4). Nevertheless, in type 2 diabetes (T2D), the macrophages persist in the M1 phenotype and cannot transport to the M2 phenotype, leading to chronic inflammation. Diabetic wounds encounter obstacles during the process of healing, resulting in wounds that tend to remain in the inflammatory state instead of entering the following proliferation phase (26). Compared to normal wounds, diabetic wounds have a dysregulated and long-lasting pro-inflammatory M1 macrophage phenotype. A study found that the population of both M1 and M2 macrophages had a considerable decrease throughout the inflammatory phase in mice with diabetes (27). During the proliferative phase, M1 macrophages in the wounds of diabetic mice significantly exceeded that in the wounds of control animals, but the levels of M2 macrophages remained relatively low. During the remodeling phase, the concentration of M1 macrophages in diabetic wounds remained significantly higher. In contrast, the concentration of M2 macrophages was comparable to that in the wounds of control mice (28). Another study discovered that the levels of the M1 markers IL-1β, nitric oxide synthase (iNOS), and TNF-α were drastically elevated in diabetic mice, whereas the M2 markers transforming growth factor-β (TGF-β), interleukins-10 (IL-10), and arginase 1 (Arg1) levels declined. Meanwhile, immunofluorescence staining found that the proportion of M1-like macrophages was notably higher in the diabetic mice than in the normal mice, while the proportion of M2-like macrophages was decreased (28). Wound macrophages from patients with type 2 diabetes (T2D) produce higher inflammatory cytokines compared to non-diabetic control persons. Macrophages are unable to complete the transition from the M1 phenotype to the M2 phenotype during the proliferative phase of wound healing (10). Plenty of investigations have demonstrated that diabetic wounds are improved by the promotion of anti-inflammatory macrophage (M2) polarization reversing macrophage-mediated inflammation. Several studies have found that astragalus polysaccharide (APS) and emodin have been shown in multiple trials to enhance diabetic wound healing by inducing M2 polarization, which reduces inflammation. The primary biologically active component of Astragali Radix, astragalus polysaccharide (APS), has an anti-inflammatory effect (29). APS stimulates the production of R-spondin3 and β-catenin while suppressing the expression of NF-κB and GSK-3β (30). This contributes to the conversion of M1 macrophages into M2 macrophages, decreasing excessive inflammation in diabetic wounds. Emodin, a derivative of anthraquinone, has many bioactivities, such as antibacterial, anticancer, and anti-inflammatory effects (31). Emodin demonstrated the ability to hinder the p65-NF-κB complex and enhance the prevalence of M2-like macrophages. Emodin treatment markedly enhanced the percentage of M2 macrophages and the expression of TGF-β, a cytokine associated with extracellular matrix (ECM) synthesis (31). Another study found that interleukins-25 (IL-25) contributed to improved diabetic wound healing by promoting the transformation of M2 macrophages and activating fibroblasts. Injection of IL-25 can improve excessive inflammation in diabetic mice, reduce vascular production, and collagen disorders, and slow wound healing (32). The distribution of M1/M2 macrophages and changes in cytokines during wound inflammation and healing can be studied in depth in the future. Employing macrophages as important regulators in wound inflammation and healing can be regarded as a viable new treatment (33).

## 4 Histone modifications regulate macrophage phenotype

**Figure 3:**
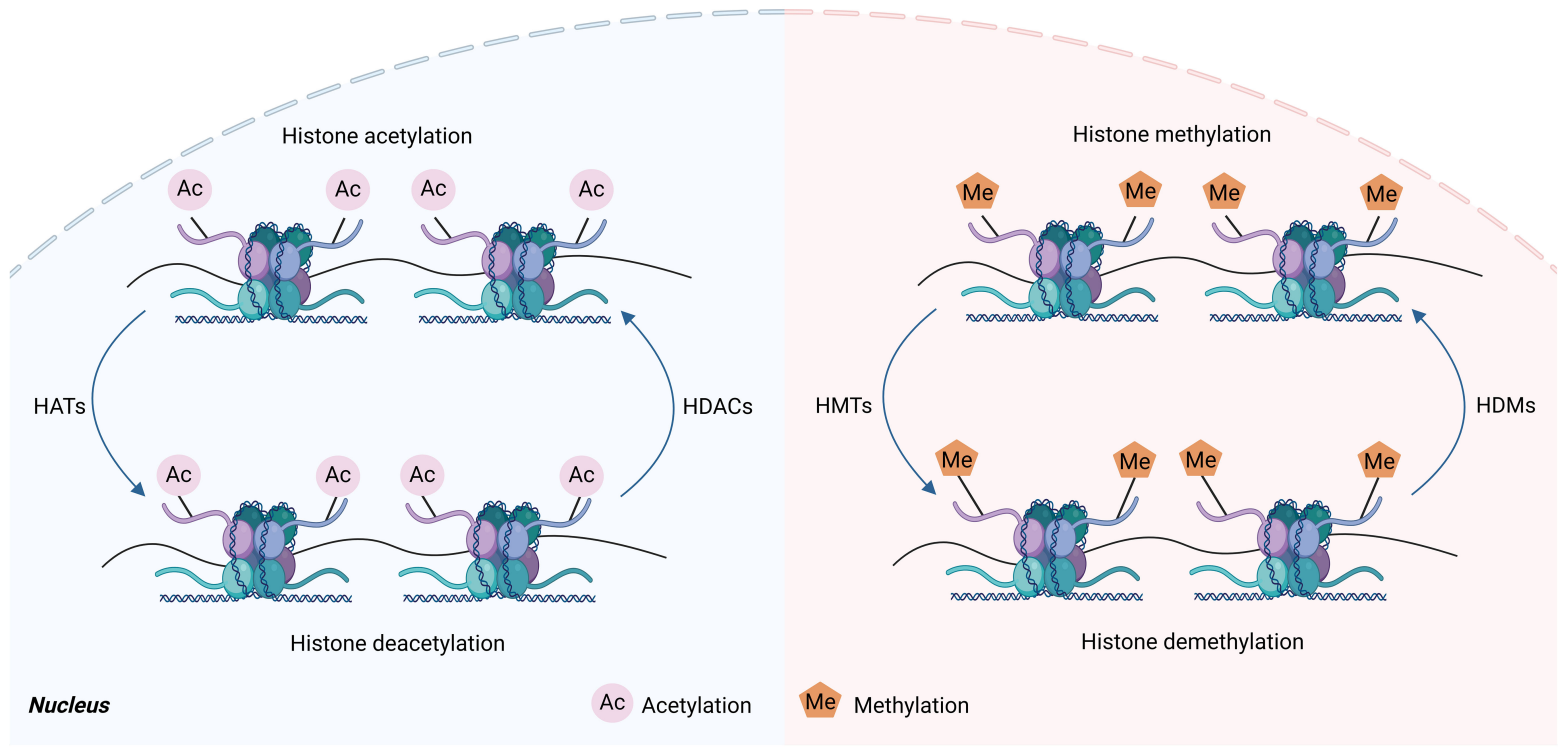
Histone acetylation and histone methylation. Histones are subjected to various post-translational modifications, such as methylation and acetylation, which are dynamically carried out by histone-modifying enzyme complexes, and these enzymes can methylate or acetylate specific residues on the histone tail. Histone methyltransferases (HMT) are mainly involved in regulating histone methylation, which transfers methyl groups to histone lysine residues, whereas histone demethylases (HDM) have the opposite effect. Histone acetyltransferases (HAT) regulate histone acetylation, which transfers acetyl groups to histone lysine residues. However, histone deacetylases (HDAC) have the opposite effect. This figure was created with the aid of Biorender (https://biorender.com/).

Multiple studies have proved that histone modifications play an essential role in controlling macrophage phenotype (10, 34, 35). Histone modification is the enzymatic process that involves altering histones through various modifications, including adenosine diphosphate ribosylation methylation, phosphorylation, acetylation, ubiquitination, and lactation (36). As a constituent of octamer, histone can undergo multiple post-translational modifications that occur via various histone-modifying enzymes (37). Histone modification can both eliminate or introduce binding sites in certain protein compounds, as well as influence the interactions between histones and DNA or other histones, changing the loose or agglutinating state of chromatin, thereby regulating gene expression (37). Enzymes involved in histone modifications, including histone demethylases (HDMs), histone acetyltransferases (HATs), histone deacetylases (HDACs), and histone methyltransferases (HMTs), are responsible for adding and removing modifications to histone (38) (**Figure 3**). Histone methylation is dependent on the precise location of methylation. Histone lysine methylation can influence transcriptional repression and activation, whereas arginine methylation increases transcriptional activation (39). Histone acetylation is commonly linked to the activation of genes by diminishing the interactions between histones and DNA, hence facilitating the access of different transcription factors to specific areas (40). Studies have demonstrated that histone modifications can affect macrophage phenotype and inflammatory gene production (5, 10).

A classic instance of an enzyme participating in histone methylation is Jmjd3, an H3K27 demethylase. Jmjd3 has an impact on macrophage polarization, and Jmjd3 inhibitor-treated macrophages showed a decrease in pro-inflammatory cytokine levels (41). Jmjd3 targeting with small molecule inhibitors reduces the ability of human primary macrophages to respond to inflammation (42). Research has demonstrated that Jmjd3-mediated H3K27 demethylation is essential for controlling the growth of M2 macrophages (35). Makoto Ishii et al. found that the epigenetic regulation of M2-macrophage marker genes involves reciprocal alterations in histone H3 lysine 27 (H3K27) and histone H3 lysine 4 (H3K4) methylation (43). Furthermore, the researchers showed that the H3K27 demethylase Jmjd3 is responsible for removing the H3K27 methylation marks (43). Persistent IL-4 treatment results in a reduction in H3K27 methylation in the promoter region of M2 marker genes, accompanied by a simultaneous upregulation of Jmjd3 expression (43). Elevated Jmjd3 leads to a reduction in H3K27 dimethylation and trimethylation (H3K27me2/H3K27me3) marks and the activation of certain M2 marker genes during transcription (43). SET and MYND domain containing 3 (Smyd3) is an H3K4 methyltransferase implicated in histone methylation and has been demonstrated to modulate M2 polarization positively (40). By stimulating the tricarboxylic acid cycle and concurrently controlling the transcriptional activities of the metabolic enzymes citrate synthase, succinate dehydrogenase complex subunit C, and pyruvate carboxylase, Smyd3 facilitates the M1-M2 conversion of macrophages (44). The histone 3 lysine 36 (H3K36) and histone 3 lysine 36 (H3K36)-specific methyltransferase known as SET and MYND domain-containing 2 (Smyd2) was shown to be a new inhibitory regulator for M1 polarization and macrophage activation (45). Increased expression of Smyd2 inhibits the synthesis of pro-inflammatory cytokines such as TNF-α and IL-6. Smyd2-mediated H3K36 dimethylation at the promoters of IL-6 and TNF-α is crucial for controlling macrophage activation during an inflammatory response (45). Irina Tikhanovich et al. discovered that arginine methyltransferase 1 (PRMT1) is crucial for M2 polarization by methylating histone H4R3me2a at the peroxisome proliferator-activated receptor γ (PPAR γ) promoter (46). PPARγ is a key transcription factor that modulates macrophage polarization toward the M2 macrophage phenotype. Setdb2 is a methyltransferase that catalyzes the trimethylation of histone 3 lysine 36 (H3K36me3) and overexpression of Setd2 suppressed M1 macrophage polarization (47).

## 5 Histone modifications of macrophages in diabetic wound healing

**Figure 4:**
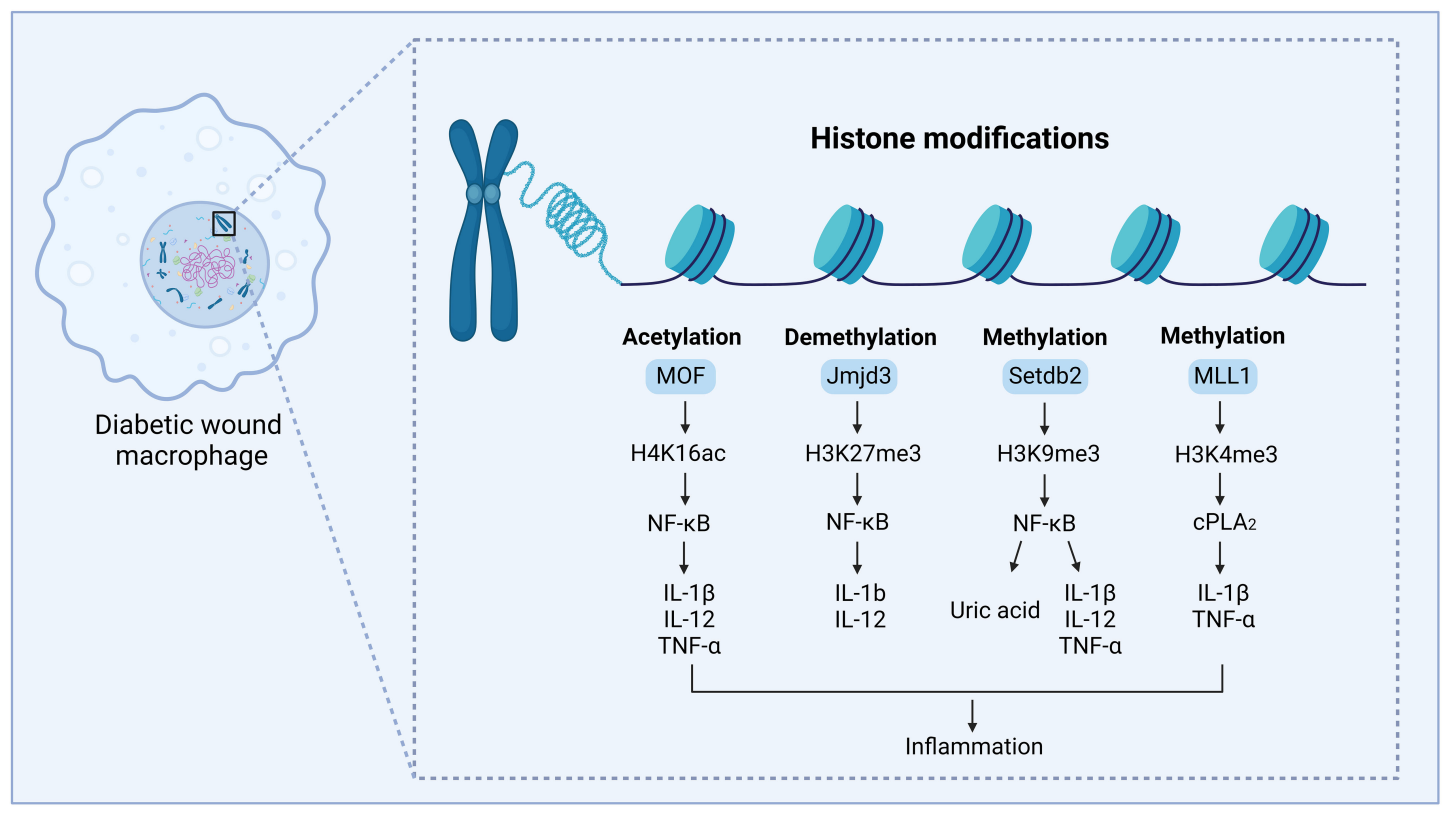
Regulation of histone modifications in diabetic wound healing, by mediating macrophage-mediated inflammation. The modification site and modification type are shown on H3 and H4. MOF can acetylate lysine 16 on the histone H4 (H4K16) and regulate NF-κB-mediated inflammatory cytokine expression, thus leading to inflammation and delayed diabetic wound healing. MLL1 can methylate lysine 4 on histone H3 (H3K4) and regulate the COX-2/PGE2 pathway, promoting the expression of inflammatory cytokines, thus leading to inflammation and delayed diabetic wound healing. Setdb2 can methylate lysine 9 on histone 3 (H3K9) and regulate NF-κB-mediated inflammatory cytokine expression and production of uric acid, thus leading to inflammation and delayed diabetic wound healing. JMJD3 can demethylate the lysine 27 site on histone 3 (H3K27) to regulate NF-κB-mediated inflammatory cytokine expression, modulating inflammation and causing delayed diabetic wound healing. This figure was created with the aid of Biorender (https://biorender.com/).

Genes are periodically repressed and activated in a cell or tissue-specific way throughout normal growth (19). Histone modifications are often enriched at certain genomic regions, especially at genes, where their presence is favorably or negatively linked with transcriptional activity (48). However, under disease conditions, histone modifications may be dysregulated and result in grave consequences (49). According to several studies, the occurrence, development, and repair of diabetic wounds are significantly influenced by the inflammatory response involving macrophages (50). Dysregulation of macrophage polarization may be linked to aberrant histone modifications. Recent research aimed at the significance of histone modification alterations in the development of illness because it influenced the dynamic modulation of macrophage phenotype. This section will primarily concentrate on the role of alterations in histone modifications for regulating the macrophage phenotype during the diabetic wound healing process (**Figure 4**; **Table 1**).

**Table 1:** Histone modifying enzyme that regulate macrophage phenotype in diabetic wound.

Types of histone modifications	Major regulator	Target histone	Function	Expression in diabetic wound	Pharmacologic inhibitors to promote the M2 phenotype	Reference
Histone methylation	MLL1	H3K4me3	Control wound macrophage function by regulating the PGE2 pathway	Upregulation	Nanocarrier containing the COX-2 inhibitor	(51)
	MLL1	H3K4me3	Early deficit and late overexpress in diabetic wound macrophages	Upregulation	MI-2	(34)
	MLL1	H3K4me3	Influence diabetic macrophage’s responsiveness to TLR4 stimulation	Upregulation	TAK-242	(52)
	Setdb2	H3K9me3	Inhibit the production of NF-κB-mediated inflammatory cytokines and XO-mediated UA in macrophages	Downregulation	NA	(10)
Histone demethylation	Jmjd3	H3K27me3	Result in the expression of inflammatory cytokines (IL-12) and lead to persistent wound inflammation	Upregulation	NA	(25)
	Jmjd3	H3K27me3	Shape macrophage toward a proinflammatory state	Upregulation	GSK-J4	(53)
	Jmjd3	H3K27me3	Regulate STING in pathologic wound macrophage	Upregulation	GSK-J1	(11)
Histone acetylation	MOF	H4K16ac	Promote NF-κB-mediated inflammatory gene transcription in diabetic wound macrophages	Upregulation	Etanercept	(54)
Histone deacetylation	HDAC3	N/A	Pharmacological blockade of HDAC3 regulates macrophage activation	Upregulation	BG45	(55)
	HDAC6	N/A	HDAC6 inhibitor inhibits tubulin-mediated IL-1β secretion	Upregulation	TSA	(56)

MLL1, mixed lineage leukemia 1; Setdb2, the histone methyltransferase; Jmjd3, Jumonji domain-containing protein 3; HDAC, histone deacetylase; MOF, the histone acetyltransferase males absent on the first; N/A, function or target not available.

### 5.1 Histone lysine methylation/demethylation in diabetic wound healing

Histone methylation is a common type of histone modification where a methyl group (-CH3) is added to a lysine or arginine residue (39). This process has the potential to change the expression of downstream proteins and exert an influence on several cellular processes. Histone methylation can enhance or inhibit transcription, relying on the targeted residue and the number of methyl groups that are introduced (57). Lysine methylation can be the addition of monomethyl (me), dimethyl (me2), or trimethyl (me3) to the ϵ-amino group (58). The histone methyltransferase known as mixed-lineage leukemia 1 (MLL1) has site specificity towards lysine 4 on histone H3 (H3K4) (59). Several research studies have found that the mechanisms mediated by MLL1 alter the phenotype of macrophages (34). Jumonji domain-containing 3 (Jmjd3) is a histone demethylase that specifically removes trimethylation of histone H3 at lysine 27 (H3K27me3). H3K27 trimethylation (H3K27me3) in gene promoter areas is linked to a compact chromatin structure, resulting in the efficient silencing of genes. Increasing the expression of Jmjd3 leads to the elimination of inhibiting histone methylation and the initiation of gene transcription (41, 60). Various research has demonstrated that increased levels of Jmjd3 in macrophages of diabetic wounds result in enhanced expression of inflammatory genes. Setdb2 is also a histone methyltransferase that trimethylates H3K9 (H3K9me3), suppresses transcription, and regulates macrophage phenotype in the process of diabetic wound healing (10). The following chapters will provide a detailed explanation of how histone methylation and histone demethylation regulate the phenotype of macrophages in diabetic wounds.

#### 5.1.1 The histone methyltransferase MLL1 directs macrophage-mediated inflammation in diabetic wound healing

MLL1 functions as a methyltransferase that enhances the levels of H3K4me3 at NF-kB binding sites in wound macrophages. This increase in H3K4me3 is linked to elevated expression of inflammatory cytokines and leads to heightened inflammation in diabetic wounds (61). Kimball et al. discovered that MLL1 is modified in a diabetic mice model (34). Compared with controls, macrophages in prediabetic mice showed a similar decrease in MLL1 and H3K4me3 levels near the promoters of inflammatory genes and inflammatory cytokines (34). Macrophages in the mice that were in the early stages of developing diabetes showed an elevation in MLL1 and H3K4me3 levels at the promoters of genes associated with inflammation, as well as inflammatory cytokines (34). Monocytes from individuals with type 2 diabetes exhibited elevated levels of MLL1 in comparison to control persons without diabetes (34). Davis et al. found that COX-2/PGE2 is elevated in diabetic macrophages, and this pathway may modulate macrophage-regulated inflammation (51). The levels of COX-2/PGE2 in wound macrophages are influenced by epigenetic modulation of crucial enzymes in the upstream pathways that regulate PGE2 production. Furthermore, MLL1-mediated H3K4 trimethylation of the cPLA2 promoter resulted in an upregulation of cPLA2 gene expression, which was observed in monocytes from people with T2D as well as in wound macrophages from diabetic mice (51). In addition to the COX-2/PGE2 pathway, Davis et al. found that the TLR4 pathway is also associated with macrophage-mediated inflammation in diabetic wounds. TLR4 expression and signaling have been observed to be markedly elevated in both diabetic people and mice (62). MLL1 promotes toll-like receptor 4 (TLR4) expression in diabetic wound macrophages by H3K4 trimethylation at the TLR4 promoter. This regulation may control the heightened inflammatory response observed in diabetic macrophages during wound healing (52). These studies indicate that MLL1 plays a crucial role in macrophage phenotypic conversion. Additionally, it may be highly relevant to macrophage-mediated inflammation in other secondary complications associated with diabetes.

#### 5.1.2 The histone demethylase Jmjd3 contributes to the inflammatory macrophage phenotype seen in diabetic wound healing

Jmjd3 is an enzyme that removes methyl groups from histone H3 at the lysine 27 site (H3K27). It is expressed in macrophages in response to inflammatory cytokines (63). Gallagher et al. discovered that alterations in histone methylation can impact gene expression in several models of T2D, and IL-12 gene expression may be altered by H3K27me3 methylation (25). In diabetic wound macrophages, Jmjd3 enhances the production of the pro-inflammatory cytokine IL-12, which can be counteracted by inhibiting Jmjd3 (25). Davis et al. demonstrate that palmitate stimulates inflammation mediated by macrophages by increasing the activity of the Jmjd3 which is one of the histone methyltransferases, and this activity relies on the TLR4/MyD88 pathway (53). In normal wound macrophages, Jmjd3-dependent histone modifications primed macrophages towards a pro-inflammatory state. When Jmjd3 is upregulated, it removes the suppressive histone methylation mark (H3K27me3) on the promoters of NF-κB-mediated inflammatory genes inducing macrophage-mediated inflammation (53). Jmjd3 can modulate inflammation in wound macrophages by reducing H3K27me3 levels on promoters of inflammatory genes through the JAK1/JAK3/STAT3 pathway (11). A study conducted by Audu et al. discovered that diabetic wound macrophages exhibit elevated levels of Jmjd3, leading to higher expression of inflammatory genes. The researchers propose that in diabetic wounds, macrophages are stimulated by IL-6, resulting in the activation of Jmjd3 and subsequent prolonged inflammation (11). This cascade sensitizes STING to reduced IFN-I and an elevated NF-κB pathway, resulting in M1 macrophage phenotype and poor wound repair in diabetes individuals (11).

#### 5.1.3 The histone methyltransferase Setdb2 modulates uric acid production and macrophage phenotype in diabetic wound healing

Diabetic wounds are characterized by chronic dysregulated inflammation and impaired tissue recovery. Macrophage plasticity, which allows macrophages to convert from a pro-inflammatory phenotype to a pro-healing phenotype, is essential for normal wound recovery (64). Prior research has demonstrated that Setdb2 plays a vital role in pro-inflammatory responses and innate and adaptive immunity (65). It achieves this by influencing type I IFN responses and regulating the expression of NF-κB target genes (66). A study found that the expression of histone methyltransferase Setdb2 differed in macrophages derived from diabetic patients’ wounds and healthy persons (10). Setdb2 functions as a regulator of macrophage polarization during the inflammatory stage of diabetic wound healing (67). It facilitates the change from the M1 phenotype to the M2 phenotype. The loss of Setdb2 is linked to a pro-inflammatory response. A specific group of pro-inflammatory genes is not normally suppressed to end the original inflammatory response, which is related to decreased H3K9 methylation at the promoters of impacted genes (67). In normal wound healing, deletion of Setdb2, specifically in myeloid cells, hindered the transformation of macrophages from pro-inflammatory states to anti-inflammatory states (10). The expression of Setdb2 failed to increase in diabetic wounds, leading to macrophages being in an inflammatory state and impairing tissue recovery (10). This is because Setdb2 methylates histone 3 with three methyl groups at NF-κB promoters to inhibit transcription (10). Setdb2 expression in wound macrophages was controlled by interferon β (IFN-β). IFN-β is a crucial type I interferon cytokine produced by the immune system. It has antiviral and antitumor properties and orchestrates the innate immune response to inflammation, tumors, and infection. In normal wound macrophages, IFN-β enhances the expression of Setdb2, leading to the observed change in macrophage phenotype during the shift to the proliferative phase. However, in diabetic conditions, the function of the IFNβ-Setdb2 pathway became impaired. The diminished IFN-β signaling in diabetic wound tissue might result in a decline in Setdb2 levels, which hinders the transformation of wound macrophages into a reparative phenotype (10). Furthermore, Setdb2 can influence the uric acid (UA) route of purine catabolism in macrophages, impacting inflammation response (10). It has been found that UA can be elevated in T2D, leading to chronic low-grade inflammation and having inflammatory influences on macrophages indirectly and directly (68). Xanthine dehydrogenase (XDH) and xanthine oxidase (XO) are the rate-limiting enzymes for the production of UA from xanthine and hypoxanthine (69). Kimball et al. discovered that the XDH promoter was simultaneously occupied by Setdb2 and NF-κB, resulting in the transcriptional suppression of XDH by Setdb2-H3K9me3 in wound macrophages (10). In addition, Kimball et al. found that diabetic macrophages showed increased xanthine oxidase and uric acid. Blocking xanthine oxidase can reduce inflammation and improve wound healing (10). Diabetic wounds can be improved by targeting Setdb2 or the UA with drugs.

In addition to Setdb2, another histone methyltransferase, SET7/9, has been proven to play a role in diabetic wound healing. SET7/9 is an enzyme responsible for adding a methyl group to histone 3 at lysine 4 (H3K4) in order to maintain the structure of euchromatin (70). SET7/9 is a crucial regulator in the regulation of hypoxia-inducible factor-1α (HIF-1α) methylation and the activation of HIF-1α target genes involved in the processes of angiogenesis and wound healing, particularly in situations of hypoxia and hyperglycemia (71). A study discovered that Set7_1a, which stabilizes HIF-1α and is an inhibitor of SET7/9 methyltransferase, can result in decreased methylation of HIF-1α at the lysine 32 residue. This leads to increased HIF-1α and the activation of genes targeted by HIF-1α that facilitate angiogenesis, such as VEGF, the glucose transporter type 1 (GLUT1), in conditions of low oxygen and high glucose levels (71). Moreover, Set7_1a can also improve wound healing in diabetic mouse models by activating the HIF-1α signaling pathway (71).

### 5.2 Histone arginine methylation/de-methylation in diabetic wound healing

Histone methylation can occur at different sites in histone, predominantly on arginine and lysine residues. It can be directed by various positive and negative regulators, even at a single site, in order to either activate or suppress transcription. Methylation can occur at different sites in histone (72). Arginine can be monomethylated or dimethylated by protein arginine methyltransferases (PRMTs) (73). Arginine residues undergo post-translational modifications by the addition of methyl groups, leading to the formation of monomethyl-arginine, asymmetrical dimethylarginine (ADMA), or symmetrical dimethylarginine (74). Methylation of arginine residues by PRMTs regulates essential physiological activities such as transcription, the DNA damage response, signal transduction cascades, and RNA processing (75), as well as the development of some diseases. Some studies found that PRMTs may play a role in inflammatory response. Protein arginine methyltransferases (PRMTs) can not only methylate histones but also methylate non-histones (73). The significance of arginine alterations in diabetes complications, whether dependent on histones or independent, is still unclear. There is compelling studies indicating that PRMT1 and CARM1/PRMT4 combine with NF-κB and add methyl groups to H3 arginine residues, thereby promoting the expression of NF-κB gene targets (76). Protein arginine methyltransferase 1 (PRMT1), coactivator-associated arginine methyltransferase (CARM1), and protein arginine methyltransferase 4 (PRMT4) are histone arginine methyltransferases. CARM1/PRMT4 is a recently identified transcriptional coactivator of NF-kB that acts as a particular regulator of NF-kB recruitment to chromatin (77). These studies indicate that arginine methylation may have a role in increasing proinflammatory responses. In addition, other studies observed that PRMT6 is recruited to NF-κB target promoters, where it presumably alters the histone code and/or methylates other chromatin-associated proteins, facilitating transcription (78). Some researchers found that p65 is demethylated on histone R30 by PRMT5, leading to the activation of NF-κB (79). This activation is accountable for the transcription of 75% of the inducible genes that encode cell-adhesion molecules, chemokines, cytokines, and kinases, and these genes, such as TNF-α, IL-8, MAP3K8, and ITGB2, play essential roles in inflammation (79). These findings above suggest that PRMTs may play a part in the inflammatory response and thereby may play a part in diabetic wound healing. However, research on histone arginine methylation in diabetic wound healing is limited and warrants further inquiry, indicating that more research is needed.

### 5.3 Histone acetylation/de-acetylation in diabetic wound healing

Histone acetyltransferases (HATs) facilitate the transfer of an acetyl group from acetyl-CoA to particular lysine residues located on histones, which is called histone acetylation (80). Histone deacetylation is catalyzed by histone deacetylases (HDACs), which enzymatically eliminate acetyl functional groups from histone lysine residues (81). HDACs can be categorized into four distinct classes: class I such as HDAC 1 and HDAC 2, class IIa such as HDAC 4 and HDAC 7, class IIb such as HDAC 6 and HDAC 10, class IV such as HDAC 11, and class III including sirtuins (82). According to certain research, diabetic foot ulcer patients had considerable downregulation of HDAC2, HDAC8, SIRT1, SIRT2, SIRT3, and SIRT7 and overexpression of HDAC1, HDAC3, HDAC4, HDAC11, and SIRT3 (83). Further, reports from Karnam et al. showed that the treatment of HDAC inhibitors can improve diabetic wound healing by regulating macrophage function and angiogenesis (56, 84). These data indicate that inhibiting histone deacetylation could be a promising target for therapy.

#### 5.3.1 HDAC inhibitors promote diabetic wound healing by regulating macrophage activation and angiogenesis

A change in macrophage activation is considered a theory for poor wound healing in patients with diabetes. The equilibrium between the M1 and the M2 macrophage phenotype is essential for the process of diabetic wound healing. Diabetic wounds have a prevalence of M1 macrophages that experience prolonged inflammatory activation, resulting in heightened expression of IL-1β. This suppresses the healing processes and leads to a decrease in M2 macrophages, along with lower growth factors, ultimately causing a delay in wound healing (84). Histone acetylation is a chromatin alteration that can be reversed and has been linked to the macrophage response to environmental signals (85). Several investigations have discovered that HDAC3 functions as an epigenomic regulator that inhibits the process of macrophage alternative activation (85). In addition, HDAC3 is crucial in facilitating the activation of the inflammatory gene expression program (55), which could be therapeutically significant in addressing inflammatory conditions such as diabetic wounds. While the regulation of macrophage function by histone changes has been extensively investigated in other inflammatory disorders, there is limited research on this topic in the context of diabetic wounds. Karnam et al. have shown that the level of HDAC3 expression is elevated in diabetic mice wounds in comparison to healthy mice (55). The selective inhibitor of HDAC3 enhances the repair of diabetic wounds by controlling the activation of macrophages and angiogenesis (55). Another study discovered that the expression of HDAC6 is increased in macrophages that are exposed to high levels of glucose and in wounds of mice with diabetes. The acceleration of wound repair in diabetic mice was achieved by inhibiting HDAC6, which resulted in a reduction in tubulin-mediated exocytosis of IL-1β without affecting the maturation process. Additionally, the expression of IL-10 was raised (84). These findings indicate that HDAC6 and HDAC3 could potentially serve as novel therapeutic targets for treating diabetic wounds.

#### 5.3.2 The histone acetyltransferase MOF regulates macrophage-specific functions in diabetic wound healing

Histone H4 lysine 16 acetylation (H4K16ac), which is modulated by the histone acetyltransferase MOF, plays a vital role in the interaction of chromatin and gene expression (86). Prior research has established that the epigenetic regulation of macrophages is responsible for controlling chronic inflammation in diabetic wounds. According to some research, MOF controls macrophage-mediated inflammation in normal and diabetic wound healing. In wound macrophages, MOF controls the transcription of inflammatory genes controlled by NF-κB through an H4K16ac mechanism, leading to an increase in the production of inflammatory cytokines (54). In addition, it has been observed that MOF is abnormally increased in macrophages found in diabetic wounds (54). The increased production of MOF in macrophages during the later stages of diabetic wounds hinders the resolution of inflammation in both mice and humans. These data indicate that targeting MOF could be a feasible therapeutic approach for controlling inflammation in diabetic wounds. However, apart from this limited research, there is a scarcity of information on histone acetylation in diabetes and diabetic wound healing.

## 6 Pharmacological modulators targeting histone modification enzymes to influence macrophage phenotype

**Figure 5:**
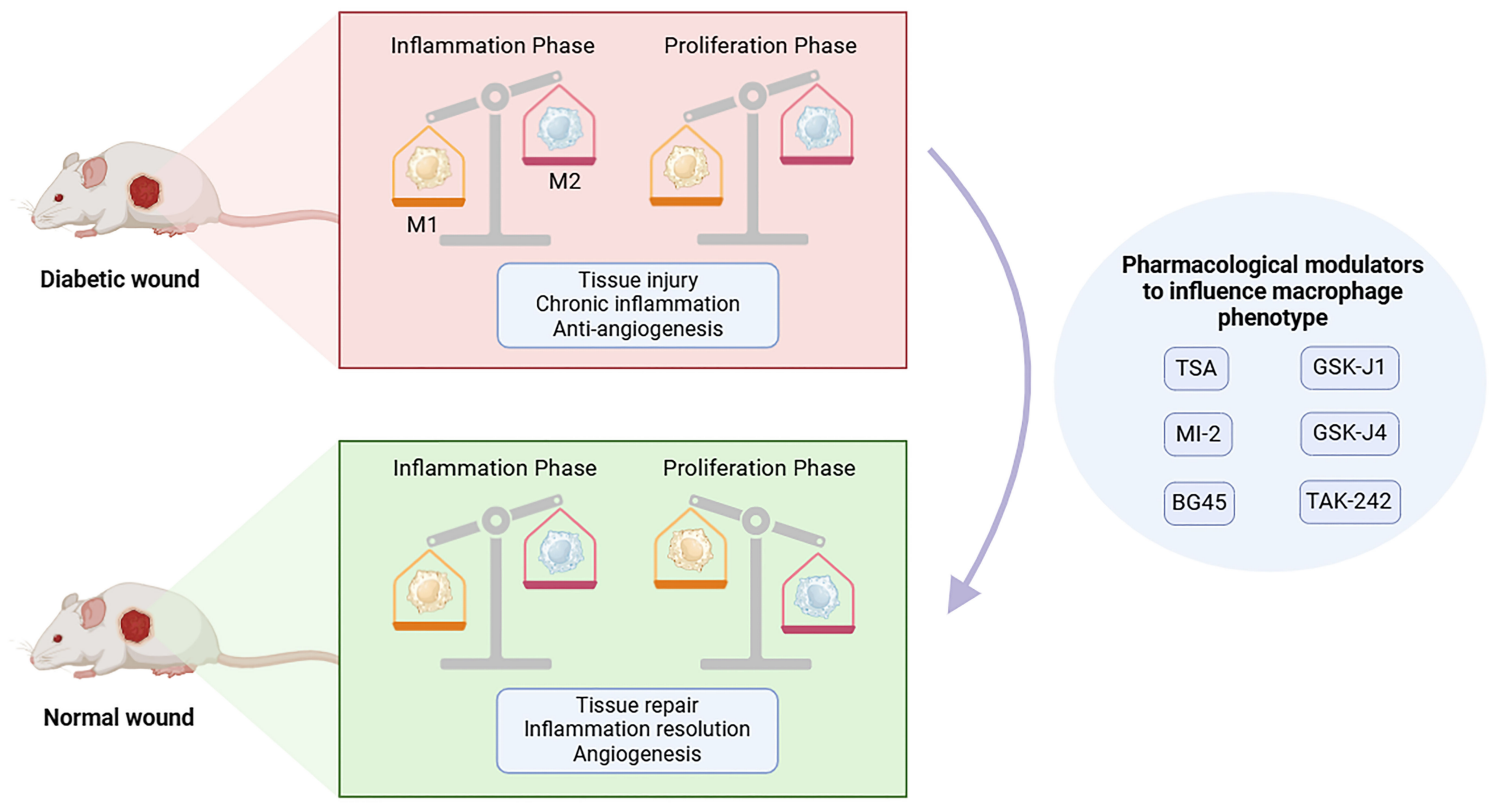
Pharmacological modulators targeting histone modification enzymes to influence macrophage phenotype. During the normal wound healing process, the phenotype of macrophages gradually shifts from pro-inflammatory M1 phenotype to anti-inflammatory M2 phenotype. In the process of wound healing in diabetes, macrophages persist in the M1 phenotype, but will not change into the M2 anti-inflammatory phenotype, which is considered to result in dysregulated angiogenesis, chronic inflammation and impaired tissue repair. The regulatory enzymes involved in histone modifications affecting macrophage phenotype may provide a new therapeutic strategy for wound healing. This figure was created with the aid of Biorender (https://biorender.com/).

In summary, we discussed how histone modifications may, to some extent, affect the expression of genes involved in the pathophysiology of diabetic wounds. Recent research has indicated that focusing on histone modifications could potentially eliminate harmful epigenetic markers and modulate the expression of genes related to diabetic wounds. Several medicines and chemicals that specifically target histone modifications have been suggested as potential treatments for this illness (**Figure 5**).

### 6.1 HDAC inhibitors accelerate diabetic wound healing by regulating macrophage activation and angiogenesis

Delayed wound healing in individuals with diabetes is characterized by prolonged activation of the inflammasome and heightened production of IL-1β in macrophages (87). Discovering and confirming new pathways that control the expression of IL-1β will offer potential treatment options for diabetic wounds. Several studies have indicated that histone deacetylase 6 (HDAC6) is increased in macrophages that are exposed to high levels of glucose, as well as in wounds of diabetic mice. The application of an HDAC6 inhibitor called Tubastatin A (TSA) gel on diabetic wounds has been found to enhance the healing process in a diabetic mice model. This is achieved by reducing the release of IL-1β through tubulin-mediated exocytosis without interfering with the maturation process. The study proposed that continuous expression of HDAC6 in diabetic wounds hinders the process of wound healing, indicating that HDAC6 could serve as a promising therapeutic target for treating diabetic wounds (84). Moreover, some research has examined the function of HDAC3 inhibitor (BG45) in macrophages stimulated by high glucose and in diabetic mice. The expression of HDAC3 is increased in diabetic mice. Using a gel containing the selective inhibitor BG45, which targets HDAC3, enhances wound healing by controlling the activation of macrophages, angiogenesis, and the levels of IL-1β (55). In conclusion, these above findings suggest that HDACs could be viable targets for therapeutic intervention in the treatment of diabetic wounds. Potential advancements in medical technology may lead to the development of diverse formulations, such as sprays, specifically designed to enhance the treatment of diabetic foot ulcers in clinical settings.

### 6.2 Histone methyltransferase and demethylase modulate macrophage phenotype in diabetic wound healing

The transition of wound macrophage response from a pro-inflammatory state to an anti-inflammatory state is a crucial element of the normal healing process (88). It is essential for the successful closure of wounds. The numerous studies demonstrating enhanced healing in diabetes patients through the reversal of chronic inflammation mediated by macrophages highlight the crucial function these cells play in wound repair in living organisms (89). A study discovered that inhibiting the histone demethylase Jmjd3, specifically in macrophages using nanoparticles, greatly enhances the healing of diabetic wounds. This improvement is achieved by reducing the levels of inflammatory cytokines and STING. These findings indicate that targeting Jmjd3 in a localized and macrophage-specific manner could be a successful therapeutic approach for promoting diabetic wounds. In addition, the histone methyltransferase MLL1 plays a role in regulating inflammation mediated by macrophages during wound healing. Furthermore, it is found to be modified in a mouse model of type 2 diabetes (34). The study indicated that the MLL1 small-molecule inhibitor, MI-2, could be an effective therapeutic medication for reducing chronic inflammation in diabetic wounds if provided at the right time after injury (34). A study discovered that Set7_1a, which contains acetonitrile (ACN) ligands, acts as an inhibitor of SET7/9 and stabilizes HIF-1α. This compound was reported to enhance wound healing in diabetic mice models by activating the HIF-1α signaling pathway and promoting the production of proangiogenic factors (71). This iridium compound is the first of its kind to target homocysteine and act as a SET7/9 antagonist specifically (71). This discovery provides a significant opportunity for the development of a new treatment for diabetic wounds by targeting and blocking the activity of SET7/9 lysine methyltransferase.

Besides, another study demonstrated that the histone methyltransferase Setdb2 influences the characteristics of macrophages and the formation of uric acid during diabetic wounds. Moreover, this study demonstrated that Setdb2 is controlled as a regulator to change macrophage phenotype both in normal and diabetic wounds through the IFNβ/JAK/STAT1 signaling pathway. They also discovered two additional factors that hinder wound healing in diabetes: the inability of wound tissue to experience a rapid increase in IFNβ and elevated UA generated by wound macrophages (10). Therefore, modulating this pathway in wound macrophages by inhibiting downstream xanthine oxidase or utilizing IFNβ-caused enhanced expression of Setdb2 holds potential as a translational therapy (10). This study identified xanthine oxidase inhibitors as a promising therapeutic approach for enhancing wound healing in diabetic mice (10). Additionally, it is stated that the IFN-I/JAK/STAT1 signaling pathway can be used to stimulate Setdb2 directly and that local delivery of recombinant IFNβ is a potential approach to treat diabetic wounds (10). Nevertheless, it is crucial to highlight the importance of the timing of administering this potential treatment. The findings of this study indicate that the timing is critical, and the administration should not take place too early as it may disrupt the essential inflammatory response required for safeguarding the host’s defense (10).

### 6.3 TNF-α inhibitor reduces MOF in diabetic wound macrophages and improves diabetic wound healing

Macrophages exhibit a chronic inflammatory state in diabetic wounds. The production of inflammatory cytokines, such as TNF-α is increased. A study discovered that TNF-α might enhance the expression of histone acetyltransferase MOF. Moreover, giving diabetic mice etanercept, an FDA-approved TNF-α inhibitor, reduced MOF levels and improved wound healing (54). The recent approval by the FDA of TNF inhibitor treatments makes it possible to target MOF through TNF-α as a potential therapeutic approach to control the chronic inflammation in diabetic wounds (54).

## 7 Conclusions and perspective

With the advancement of technology, a growing multitude of histone modifications have been discovered and proved to be essential to maintain the homeostasis of the macrophage phenotype and regulate inflammation in diabetic wounds. Most of the current research on histone modifications in diabetic wound healing is concentrated primarily on histone methylation and histone acetylation. However, the relevant research on other histone modifications, such as histone lactylation and histone phosphorylation, still needs to be completed. It was discovered that lactate and histone lactylation have a positive effect on driving M2-like gene expression during M1 macrophage polarization (90). However, it is uncertain whether histone lactylation has a role in diabetic wounds. This suggests that the current understanding of the mechanisms of histone modifications to regulate macrophage phenotypic conversion in diabetic wounds needs to be further developed.

Macrophages are important immune cells that regulate wound inflammation, but wound healing is a complicated process involving the inter-regulation of various types of cells. Current research is mainly focused on macrophages, and studies on histone modifications affecting diabetic wound healing in other cells are extremely limited. Wolf et al. found that the expression of IFN-κ in diabetic wound keratinocytes was controlled by histone methyltransferase MLL1 and that the administration of IFN-κ improved the healing of diabetic wounds (91). Future studies could also further explore how histone modifications regulate other cells and thus improve diabetic wounds. The application of therapeutic compounds that can directly disrupt the functioning of macrophages has unquestionably progressed. These compounds disrupt the function of M1 macrophages or enhance their transformation into the M2 phenotype. Although the administration of pharmacological molecules can reduce inflammation and promote diabetic wound healing, their impact on cells is not limited to macrophages, and they may also have an impact on cells other than macrophages. Targeting histone-modifying enzymes in diabetic wounds in a macrophage-specific manner may ameliorate inflammation, making it a viable therapeutic strategy to improve diabetic wounds.

In conclusion, alterations in histone modification patterns in diabetic wound macrophages trigger several downstream molecular disorders by controlling the transcription and translation of genes associated with inflammatory responses in wound healing. These downstream molecules exert their effects on cells or the extracellular matrix in the wound, ultimately resulting in obstructing diabetic wound healing. Future research could delve into the role of novel histone modifications in diabetic wounds and their influence on various types of cells. Targeting macrophages by manipulating them with nanomedicines to participate in the inflammatory response is a viable approach for diabetic wounds.
